# Drug interactions in the elderly HIV-infected patient

**DOI:** 10.1186/1758-2652-13-S4-P108

**Published:** 2010-11-08

**Authors:** C Coroiu, F Tollinchi, G Hittinger, S Mokhtari, P Granet, O Faucher-Zaegel, A Madrid, A Menard, JL Mattei, S Chadapaud, VISAGE study group

**Affiliations:** 1 CHU Conception APHM, Marseille, France; 2 Hopital St Joseph, Marseille, France; 3 CH Toulon Font pre, Toulon, France; 4 CHU Nord APHM, Marseille, France; 5 CH Digne, Digne les bains, France; 6 CHU Sud, Marseille, France; 7 CHU Conception, Marseille, France; 8 CH Toulon, Toulon, France; 9 VISAGE study group, Region PACA, France

## 

Elderly HIV-infected patients may present particularities on both disease evolution and morbidity, as compared to younger patients. Moreover, elderly patients are more likely to take numerous medications due to their age-related condition. VISAGE is a French multidisciplinary study group focusing on elderly HIV-infected patients in order to evaluate and improve their therapeutic care. Our study "Visage 1" aimed to analyse potential drug interactions between antiretroviral drugs (ARV) and all other products taken by elderly HIV-infected patients.

This 6-month prospective study involved patients treated for HIV infection and aging more than sixty years at the time of the study. Patients were to fill an anonymous self-questionnaire for reporting their ARV, diseases other than HIV infection, and drugs or products regularly taken other than ARV. Observance has been evaluated using a visual analog scale graduated on 10. Analysis of drug interactions relied on the French drug agency (Afssaps) thesaurus by using the tool available on the website www.theriaque.org.

25 women and 70 men filled 95 questionnaires. Median age was 65.3±5.2 years. Treatment of HIV infection was a combination of three ARV for 85% of patients. Among patients, 94% had concomitant treatment with non-ARV drugs (4.6 ± 3.3 drugs /patient) mainly prescribed for a cardiovascular mean. Most frequently used concomitant drugs were paracetamol, lysine acetyl salicylate, bromazepam, rosuvastatin, and zolpidem. Other products widely used were sexual stimulants and vitamins. Consumption of alcohol, poppers and cannabis occurred in 40, 3, and 2 patients, respectively. Clinically relevant drug interaction occurred in 45% of prescriptions and involved non-ARV drugs but were not in majority classified as serious. Two associations were found contra-indicated: ritonavir+alfusozine and protease inhibitor+simvastatin. Observance reached 9.9 ± 0.4 for antiretroviral drugs, and 9.8 ± 0.6 for the other drugs.

Drug interactions were less frequent and less severe than expected in this population. Physicians' awareness of concomitant drugs taken by the patient is crucial since most clinically and severe interactions occurred between ARV and non-ARV drugs. Observance was extremely high as compared with the rate usually described for the general population of HIV-infected patients and reasons of that high rate need to be further investigated.

